# Persistent and Episodic Depressive Symptoms Among Family Caregivers and Non-Caregivers in Singapore

**DOI:** 10.1093/geroni/igaf122.217

**Published:** 2025-12-31

**Authors:** Jeremy Lim-Soh, Ha-Linh Quach, Rahul Malhotra

**Affiliations:** Duke-NUS Medical School, Singapore, Singapore; The University of Sydney, Sydney, New South Wales, Australia; Duke-NUS Medical School, Singapore, Singapore

## Abstract

While family caregivers, versus non-caregivers, have a higher risk of adverse psychological outcomes, less is known about the persistence of this risk over time. We assessed (a) the risk of persistent and episodic clinically relevant depressive symptoms (CRDS; 11-item CES-D score of seven or above) in family caregivers of older adults, relative to non-caregivers, and (b) among caregivers, care-recipient and caregiver characteristics associated with such depressive symptoms. Using longitudinal data (4 waves; 6-12 months apart) on 218 caregivers and 174 non-caregivers in Singapore, we classified them into three categories of CRDS over time: (1) persistent CRDS (CRDS in two or more consecutive waves), (2) episodic CRDS (CRDS in one or two non-consecutive waves), and (3) no CRDS. The prevalence of persistent and episodic CRDS was much higher among caregivers (23.4% and 23.4%, respectively) versus non-caregivers (9.2% and 16.1%, respectively). Logistic regression, adjusting for confounders, confirmed the higher risk of caregivers, versus non-caregivers, for persistent or episodic (versus no) CRDS. Among caregivers, their own chronic diseases and their care-recipient’s mood, behavior, and memory impairments were associated with persistent (versus no) CRDS. Our findings highlight the substantial and enduring psychological distress that family caregivers are exposed to, underscoring the need to support their mental well-being.

